# Supplementary material for: Numerical and Experimental Analyses of Three-Dimensional Unsteady Flow around a Micro-Pillar Subjected to Rotational Vibration
Source: Micromachines (Basel). 2018 Dec 17;9(12):668. doi: 10.3390/mi9120668 (PMC6316184; doi:10.3390/mi9120668)
Supplement: Supplementary file 1 [file micromachines-09-00668-s001.zip › micromachines-393687 - supplementary - for final check/micromachines-393687 - supplementary - for final check.docx]

Supplementary Materials: Numerical and Experimental Analyses of Three-Dimensional Unsteady Flow around a Micro-Pillar Subjected to Rotational Vibration

Kanji Kaneko, Takayuki Osawa, Yukinori Kametani, Takeshi Hayakawa, Yosuke Hasegawa and Hiroaki Suzuki

Supplementary Text

Governing Equations of Fluid in a Moving Frame with Rotational Vibration

We introduce a new coordinate system 𝒙^∗^ = (𝑥^∗^, *y*^∗^, *z*^∗^) which moves with the same speed of the substrate:

| $\boldsymbol{x}^{*}= \left( \begin{matrix} x^{*} \\ y^{*} \\ z^{*} \end{matrix} \right) = \left( \begin{matrix} \underline{x}^{*} \\ \underline{y}^{*} \\ \underline{z}^{*} \end{matrix} \right) - \left( \begin{matrix} x_{R}^{*} \\ 0 \\ z_{R}^{*} \end{matrix} \right).$ | (S1) |
| --- | --- |

Accordingly, the relative fluid velocity to the substrate is given by

| $\boldsymbol{u}^{*}= \left( \begin{matrix} u^{*} \\ v^{*} \\ w^{*} \end{matrix} \right) = \left( \begin{matrix} \underline{u}^{*} \\ \underline{v}^{*} \\ \underline{w}^{*} \end{matrix} \right) - \left( \begin{matrix} u_{R}^{*} \\ 0 \\ w_{R}^{*} \end{matrix} \right),$ | (S2) |
| --- | --- |

where a variable without under-bar indicates a quantity in the moving coordinate. By converting from *x_i_* to *x_i_*, and from *u_i_* to *u_i_*, the governing Equations (1) and (2) can be written in the moving coordinate as

| $\frac{\partial u_{i}^{*}}{\partial t^{*}}+u_{j}^{*}\frac{\partial u_{i}^{*}}{\partial x_{j}^{*}} = -\frac{1}{\rho^{*}}\frac{\partial p^{*}}{\partial x_{i}^{*}}+\nu^{*}\frac{\partial^{2}u_{i}^{*}}{\partial x_{j}^{*}\partial x_{j}^{*}}+ f_{i}^{*},$ | (S3) |
| --- | --- |

And

| $\frac{\partial u_{i}^{*}}{\partial x_{i}^{*}} = 0,$ | (S4) |
| --- | --- |

where *f_i_* is the inertia force due to the acceleration/deceleration of the moving coordinate. It can be written as

| $\boldsymbol{f}^{*}= -\frac{d\boldsymbol{u}_{R}^{*}}{dt}= -\left( \begin{matrix} \frac{du_{R}^{*}}{dt} \\ \frac{dv_{R}^{*}}{dt} \\ \frac{dw_{R}^{*}}{dt} \end{matrix} \right)= \left( \begin{matrix} A^{*}\left( \frac{2\pi}{T^{*}} \right)^{2}\cos\left( \frac{2\pi t}{T^{*}} \right) \\ 0 \\ A^{*}\left( \frac{2\pi}{T^{*}} \right)^{2}\sin\left( \frac{2\pi t}{T^{*}} \right) \end{matrix} \right).$ | (S5) |
| --- | --- |

In order to generalize the problem, we normalize physical variables with the length scale of *δ*^*^ and the oscillation period of *T*^*^. Accordingly, the velocity-scale for normalization is given by *U*^*^*_ref_* = *δ*^*^/*T*^*^. The dimensionless coordinate is given as

| $\boldsymbol{x} \equiv\frac{\boldsymbol{x}^{*}}{\delta^{*}}= \frac{1}{\delta^{*}}\left( \begin{matrix} x^{*} \\ y^{*} \\ z^{*} \end{matrix} \right).$ | (S6) |
| --- | --- |

The velocity components are also non-dimensionalized as

| $\boldsymbol{u} \equiv\frac{\boldsymbol{u}^{*}}{U_{ref}^{*}}= \frac{T^{*}}{\delta^{*}}\left( \begin{matrix} u^{*} \\ v^{*} \\ w^{*} \end{matrix} \right).$ | (S7) |
| --- | --- |

The pressure is normalized by *p* = *p*^*^/(*ρ*^*^ *U*^*^*_ref_* ^2^) and the dimensionless time is defined as *t* = *t*^*^/*T*^*^.

Substituting the above relationships, the dimensionless forms of Equations (1) and (2) can be obtained as

| $\frac{\boldsymbol{\partial}\boldsymbol{u}_{\boldsymbol{i}}}{\boldsymbol{\partial t}}\boldsymbol{+}\boldsymbol{u}_{\boldsymbol{j}}\frac{\boldsymbol{\partial}\boldsymbol{u}_{\boldsymbol{i}}}{\boldsymbol{\partial}\boldsymbol{x}_{\boldsymbol{j}}}\boldsymbol{= -}\frac{\boldsymbol{\partial p}}{\boldsymbol{\partial}\boldsymbol{x}_{\boldsymbol{i}}}\boldsymbol{+}\frac{\boldsymbol{1}}{\boldsymbol{Re}}\frac{\boldsymbol{\partial}^{\boldsymbol{2}}\boldsymbol{u}_{\boldsymbol{i}}}{\boldsymbol{\partial}\boldsymbol{x}_{\boldsymbol{j}}\boldsymbol{\partial}\boldsymbol{x}_{\boldsymbol{j}}}\boldsymbol{+}\boldsymbol{f}_{\boldsymbol{i}}\boldsymbol{,}$ | (S8) |
| --- | --- |
| $\frac{\boldsymbol{\partial}\boldsymbol{u}_{\boldsymbol{i}}}{\boldsymbol{\partial}\boldsymbol{x}_{\boldsymbol{i}}}\boldsymbol{= 0,}$ | (S9) |

where the dimensionless inertia force *f_i_* is given by

| $f_{i}= \frac{2\pi}{St}\left( \begin{matrix} \cos\left( 2\pi t \right) \\ 0 \\ \sin\left( 2\pi t \right) \end{matrix} \right).$ | (S10) |
| --- | --- |

This way, we obtain the dimensionless governing equations (5-7) for a fluid in the moving frame.


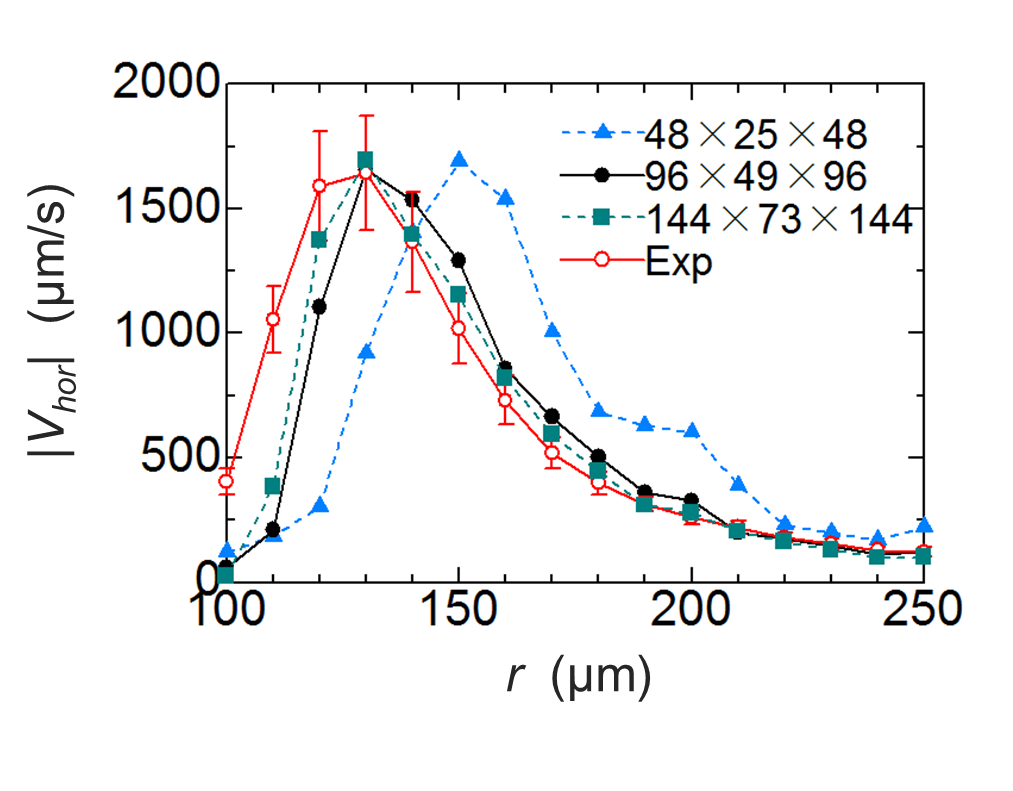


**Figure S1.** Comparison with the radial profile of |*V_hor_*| calculated with different grid resolutions in the numerical simulation. The distribution calculated with a low-resolution grid (48 × 25 × 48) differ significantly, but those calculated with medium (96 × 49 × 96; used in the main results) and high-resolutions (144 × 73 × 144) were similar.


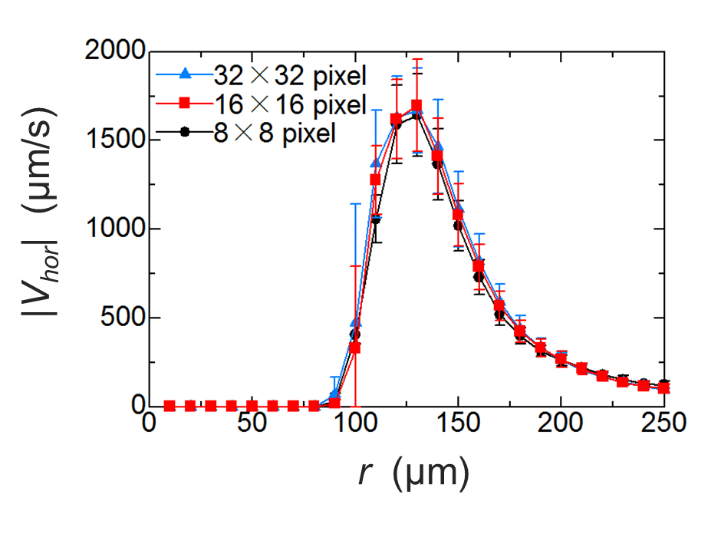


**Figure S2.** Comparison of the radial profile of |*V_hor_*| calculated with different window size in PIV analysis. One pixel in images corresponds to 0.8 μm.
